# Evolution of pathogenicity in obligate fungal pathogens and allied genera

**DOI:** 10.7717/peerj.13794

**Published:** 2022-08-25

**Authors:** Moytri RoyChowdhury, Jake Sternhagen, Ya Xin, Binghai Lou, Xiaobai Li, Chunnan Li

**Affiliations:** 1 Infectious Diseases Program, California Department of Public Health, Richmond, California, United States of America; 2 Riverside School of Medicine, University of California, Riverside, Riverside, CA, United States of America; 3 Hangzhou Academy of Agricultural Sciences, Hangzhou, P.R. China; 4 Guangxi Academy of Specialty Crops, Guilin, Guangxi, P.R. China; 5 Zhejiang Academy of Agricultural Sciences, Hangzhou, P.R. China; 6 Hangzhou Academy of Agricultural Sciences, Hangzhou, P.R. China

**Keywords:** Obligate Pathogens, Parasitism, Virulance, Pathogenicity, Biotrophy, Evolution

## Abstract

Obligate fungal pathogens (ascomycetes and basidiomycetes) and oomycetes are known to cause diseases in cereal crop plants. They feed on living cells and most of them have learned to bypass the host immune machinery. This paper discusses some of the factors that are associated with pathogenicity drawing examples from ascomycetes, basidiomycetes and oomycetes, with respect to their manifestation in crop plants. The comparisons have revealed a striking similarity in the three groups suggesting convergent pathways that have arisen from three lineages independently leading to an obligate lifestyle. This review has been written with the intent, that new information on adaptation strategies of biotrophs, modifications in pathogenicity strategies and population dynamics will improve current strategies for breeding with stable resistance.

## Introduction

**Figure 1:**
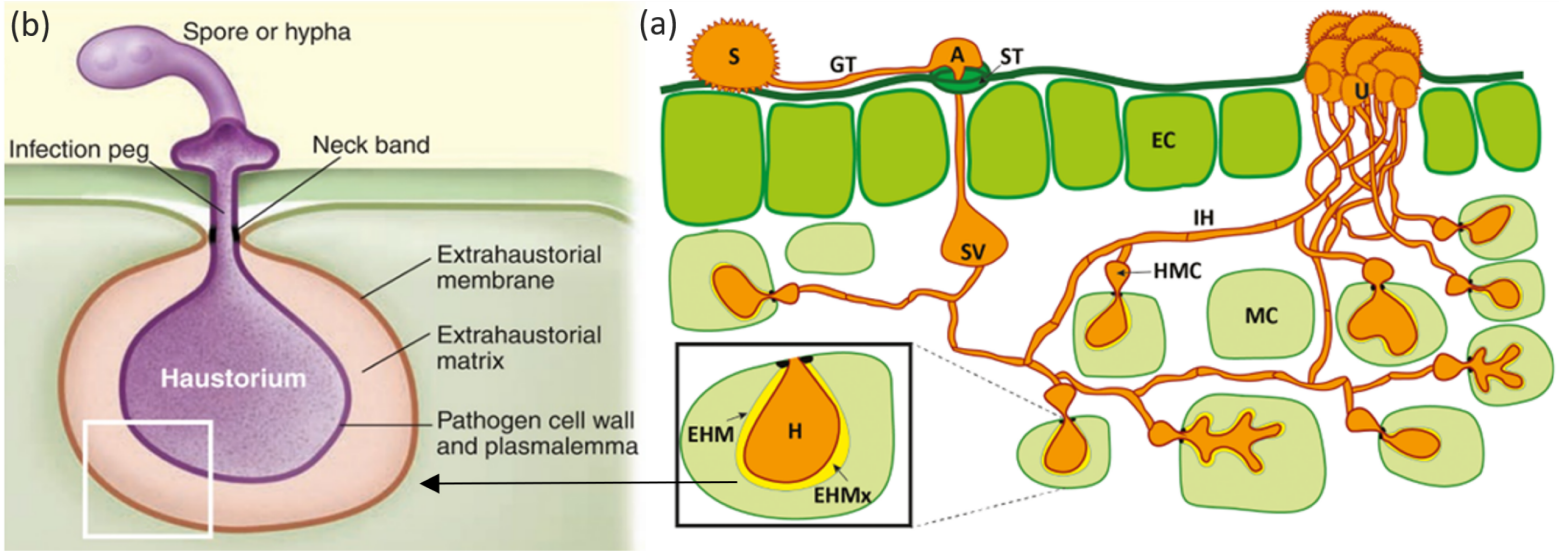
Mode of infection of an obligate pathogen (*Puccinia* sp). (A) The dikaryotic uredospore (S) lands on the leaf surface and produces a germination tube (GT) within 6 hours. Subsequently, it produces an appresorium (A) over the stomatal aperture and enters into the leaf interior through the stoma (ST), where it differentiates into a substomatal vesicle (SV). Primary infection hyphae (IH) propagate through the leaf, and once in contact with mesophyll cells, haustorial mother cells (HMC) differentiate. These penetrate the host mesophyll cell (MC) wall to form the haustorium (H). The haustorium remains separated from the host cell cytoplasm by the extrahaustorial matrix (EHMx) and the host-derived extrahaustorial membrane (EHM). After the establishment of the first haustorium, secondary hyphae develop, colonize the intercellular spaces, and give rise to more HMCs and haustoria. The cycle is completed within 10–11 days when the invasive hyphae form sporogenous basal cells in the uredia (U) and thousands of new infective uredospores erupt through the leaf epidermis. Figure modified from Garnica et al. (2014). (B) Haustorium showing a neck ring that seals the interface between the pathogen and host plasma membrane, disconnecting the extrahaustorial matrix from the plant and fungal apoplast and establishing a biotroph-specific compartment Figure has been taken from Dodds (2009).

Obligate parasites, including filamentous eukaryotes and certain oomycetes, are known to infect plants where they interact and co-evolve. The main characteristic of obligate parasites that differentiates them from other parasites is their inability to survive without a host. As an adaptive measure for survival, they are known to grow asymptomatically. Symptoms are seen during reproduction either when there is rupture of spores through the epidermis or when conidiophores on the leaves of the host plant make entry through stomatal openings (Fig. 1A). These obligate pathogens have modified themselves to reproduce asexually and/or through a sexual cycle, either on the same or different host plants, as seen in some rust fungi.

Obligate parasites influence host behavior and fitness. Usually, the modifications are mostly to the advantage of the parasite, but sometimes the modifications do not have consequences on the host or the parasite. However, infection by a parasite on a host can alter trophic interactions, biodiversity, and wood webs. Hence, these parasites play an important role in shaping the community and the ecological structure. This paper discusses the evolutionary modifications of these pathogens, and it explains how they acquire and maintain virulence. Our literature review has concluded that not all obligate pathogens are equal. Some obligate pathogens have evolved to be irreversibly specialized pathogens while there are others that are reversibly pathogenic because of transposable elements. The knowledge of this evolutionary pattern of development can help lead to the production of novel strategies to combat some of these pathogens that have impacted our ecosystem.

**Figure 2:**
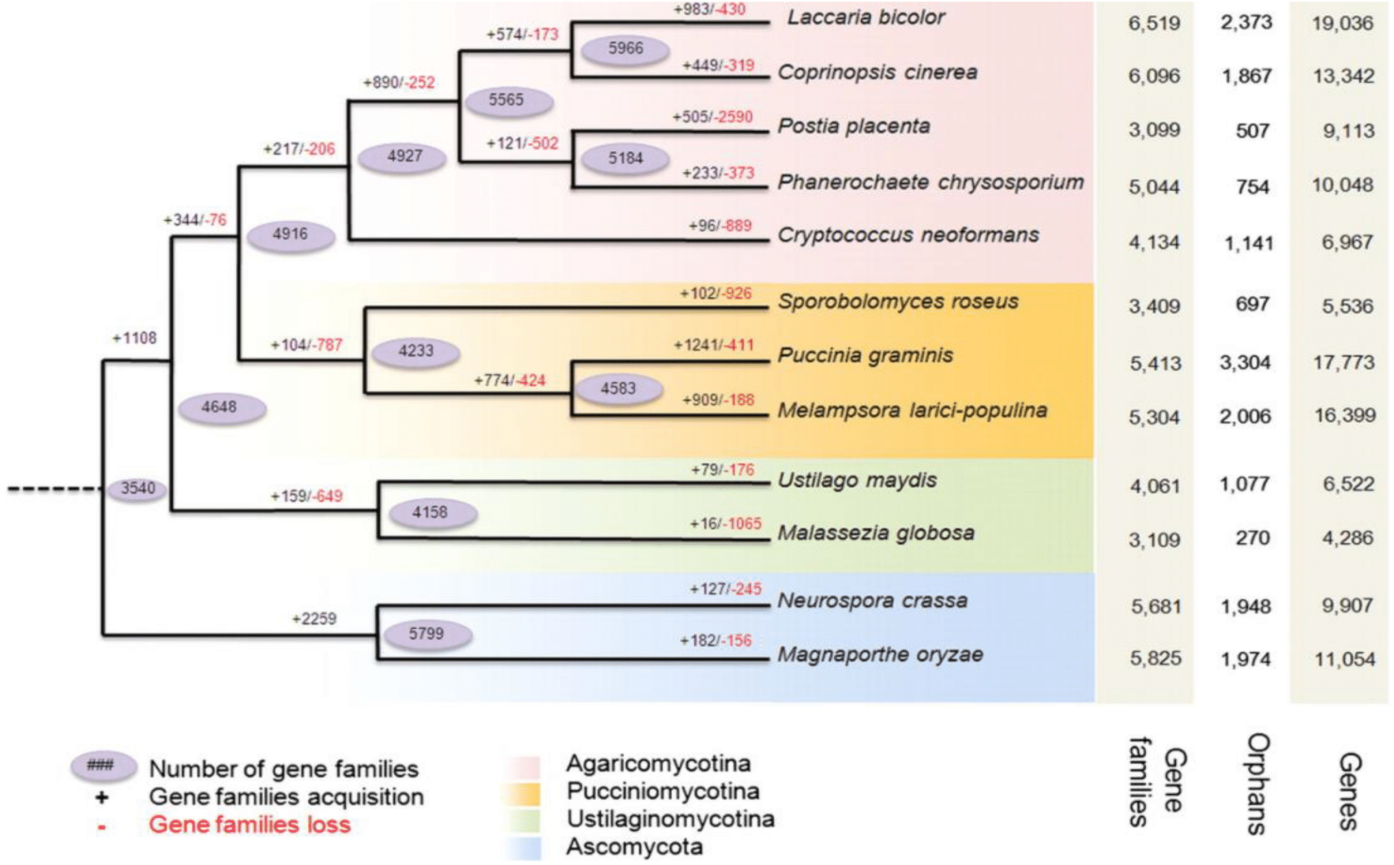
Predicted pattern of gene families gain and loss in representative fungal genomes. The figure represents the total number of protein families in each species or node estimated by the Dollo parsimony principle. The numbers on the branches of the phylogenetic tree correspond to expanded (left, black), contracted (right, red), or inferred ancestral (oval) protein families along each lineage by comparison with the putative pan-proteome. For each species, the number of gene families, orphan genes, and the total gene number are indicated on the right. Image used from Duplessis et al. (2011).

**Figure 3:**
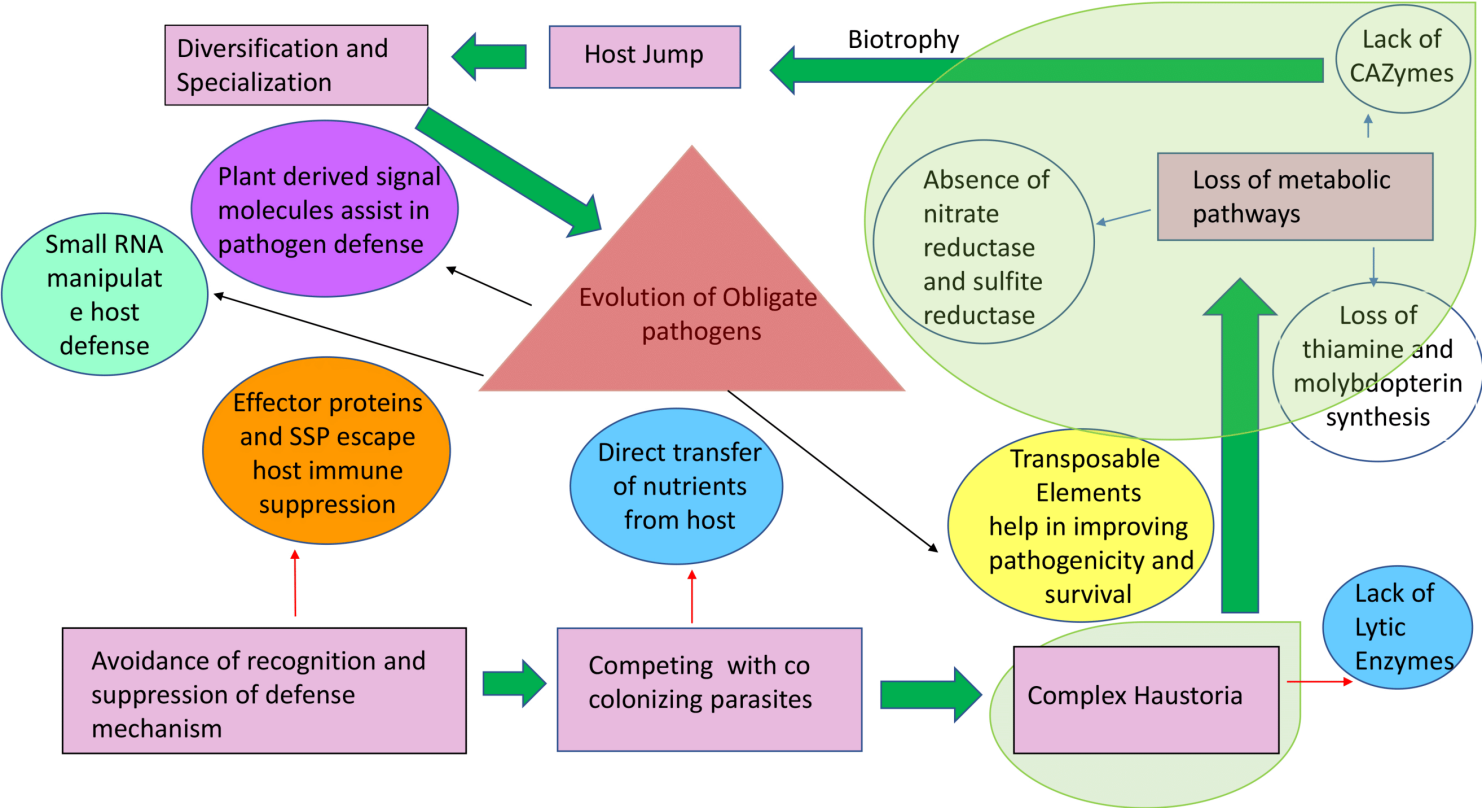
Origin and stepwise diversification of filamentous obligate biotrophic plant pathogens. Pink boxes indicate significant lifestyle changes of the pathogen during its evolution towards biotrophy. Red and blue arrows indicate movement towards genetic consequences leading to the lifestyle changes. Circles indicate other key features of biotrophy. Transparent green areas highlight the parallel evolution of the traits. The intimate association with the plant tissue is reflected in a loss of genes for pathways highlighted with blue arrows. Plant host signals transferred to the pathogen (purple circle) can drive further diversification and specialization in obligate biotrophic pathogens and effector-triggered immunity. This is an example of collateral evolution.

Characteristic features of obligate parasites are:

1. Need for a living plant tissue as a host. Several unknown factors of growth may contribute to why obligate biotrophic pathogens are unable to grow on artificial medium (Kemen, Agler & Kemen, 2015). To better demonstrate, let us use the example of dimorphic smut fungi of the order Ustilaginales. This fungus exhibits yeast-like patterns of growth on artificial media and has a biotrophic filamentous lifestyle which does not adversely affect the host (Spanu, 2012). Rust fungi were once categorized as obligate parasites, but they have undergone modifications with several species grown in axenic culture (Littlefield & Heath, 1979). Rust fungi exhibit this complicated lifestyle only on living host plants.

2. Haustoria play a necessary role in the pathogenic entry of obligate parasites through the host’s cell wall (Bozkurt, Kamoun & Lennon-Duménil, 2020). Haustoria (Figs. 1A; 1B) are formed when specialized fungal hypha penetrate the cell wall and expand inside the host cell (Szabo & Bushnell, 2001). Haustoria formation is characterized by invagination and alteration of the host’s plasma membrane. This leads to the formation of the extrahaustorial membrane, a membrane-like structure which surrounds the haustorial body (Kemen, Agler & Kemen, 2015). Haustoria allow for transportation between pathogen and host and are associated with the uptake of nutrients like sugars and amino acids. They also possess extrahaustorial matrix which assists in the transportation of mainly effector proteins in the cytoplasm of the host (Kemen, Agler & Kemen, 2015). The neck ring is a unique feature of the haustoria in obligate pathogens (Fig. 1B). This ring acts as a doorkeeper between the pathogen and the host, creating biotroph-specific compartments within the fungal apoplast (Kemen, Agler & Kemen, 2015).

3. Impaired ability to secrete cell wall-degrading enzymes and inefficient toxin production. Obligate biotrophs escape the host immune system and establish a smooth host viability to complete their life cycle. They can also recognize signals of altered host cell status as well as from the pathogen (Jones & Dangl, 2006). Rusts, which are obligate parasites, have a very limited number of genes encoding for secreted proteins and carbohydrate active enzymes (McDowell, 2011). These genes are known to kill the host cell and produce harmful signals. Low gene copy number for these traits is an evolutionary adaptation allowing for survival inside the host (Figs. 2 and 3).

The immune systems of host plants generally function by using pattern recognition receptors (PRRs) which recognize the microbe-associated molecular patterns (MAMPs) of potential pathogens. This serves as the plant’s primary immune system, establishing a basal defense response in the plant which may be suppressed by effectors from plant pathogens. Pathogen growth may then be impeded by the plant’s secondary immune system which functions by using resistance protein (RPs). Systemic resistance may be induced in the plant after the local primary and secondary immune responses are activated, allowing for the host plant to acquire resistance towards future attacks by the pathogen (de Wit, 2007). Separate pathways of defense may be activated within the plant by different infiltrating pathogens. For example, necrotrophs may activate immune responses involving ethylene and Jasmonic acid (JA), while biotrophs may activate immune responses involving salicylic acid (SA). Crosstalk between these pathways may allow for the differential recognition and response of host plants to different pathogens (Garcia-Brugger et al., 2006).

**Figure 4:**
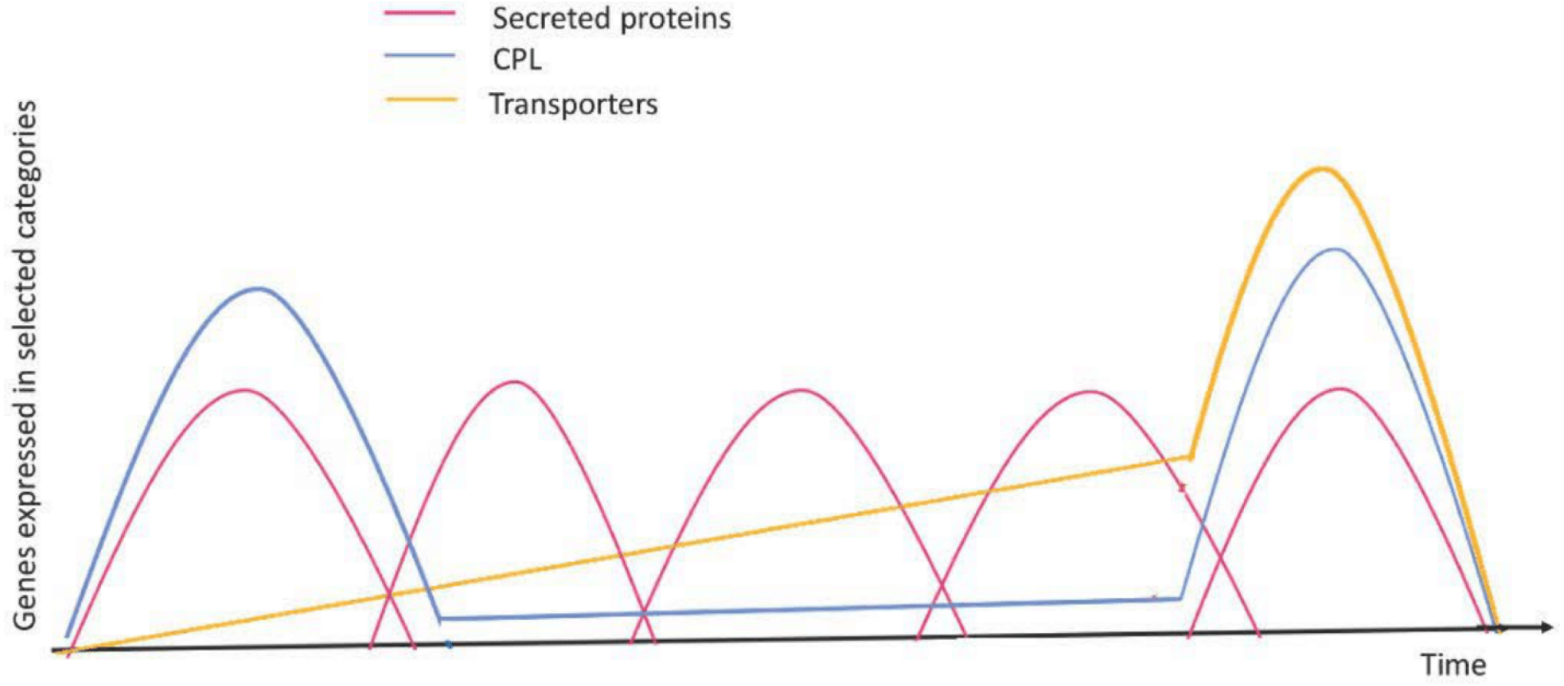
Expression profiles of selected genes during the infection process. Red curves represent coordinated waves of expression for secreted protein genes; blue curves represent the main expression profile for CAZyme, protease, and lipase (CPL) encoding genes; green curves represent expression for transporter encoding genes. Figure based on a time-course transcriptomics in Duplessis et al. (2011) and Dobon et al. (2016). Figure adapted and modified from Lorrain et al. (2019).

Chitin, a major component of the fungal cell wall, is known to serve as a signal for invasion. A high number of gene families encoding chitin deacetylases are found in Rusts and are thought to interfere with chitin surveillance (McDowell, 2011). Prior to pre-penetration in rusts there is an upregulation of serine esterases known to exhibit cutinase activity. Cutinase is the enzyme that helps in the attachment of uredospores of *Uromyces viciae-fabae* to the cuticle of the plant. The entry of the pathogen through the stomatal opening enhances chitin deacetylase activity. This enzyme protects fungal machinery inside the host from degradation and also probably helps with chitin surveillance. The formation of haustorial mother cells is associated with the synthesis of polygalacturonate lyase (PL) (Fig. 4). Using differential hybridization, cDNA was obtained for genes that were activated during later stages of infection. These genes were associated with structure differentiation of *Uromyces viciae-fabae*. The transcripts for genes *rif16* and *rif21* were observed during haustorial mother cell formation and their corresponding gene products were anticipated to be useful during infection (Deising et al., 1995). These are some of the modifications that have been employed to evade the host immune system.

4. Impaired uptake of mineral nutrients by obligate biotrophs (Duplessis et al., 2011; Tisserant et al., 2013). It has been observed that rusts’ demand for nitrogen uptake has been met by proteases which are associated with the digestion of extracellular plant proteins (McDowell, 2011). Direct nutrient uptake/transfer from the host evolved over time, allowing the pathogen to escape from competing microorganisms (Fig. 3). Millions of years of evolution led to adaptive measures to combat competition, predation, and mutualism, with respect to other microorganisms (Kemen, Agler & Kemen, 2015). The microbiome also played an important role on the host environment. As a result of new adaptations, the phyllosphere niche is affected in a number of ways. To understand how the biology of obligate biotrophic plant parasites is influenced by the microbiome, it is necessary to first understand how biotrophs evolved together and formed communities.

## Survey Methodology

We systematically searched literature databases, included the following: PubMed Advanced Search, Scopus, Institute of Scientific Information (ISI), Web of Science, and Google Scholar. A broad range of keywords and phrases were searched, including: (1) Obligate pathogens (2) Oomycetes evolution (3) Host microbe interaction (4) Parallel and Collateral evolution (5) Loss and gain of gene function in evolution (6) Convergent evolution (7) Haustoria and pathogenicity (8) Genome Wide Association (GWA) studies and pathogenicity (9) Effector proteins and virulence (10) Arms race (11) Obligate parasitism. We heavily relied on publications in the last 15 years in our study, although we referenced a few of the older publications for fundamental concepts. We also searched (Google images) for diagrams and for any schematic figures to help our understanding of fundamental concepts in the area. We did not include studies that had only abstracts available with no full text information. Our literature search did not screen papers based on date of publication, the impact factor of the journal, name of the journal, or author affiliation.

### Evolution of pathogenicity in obligate pathogens

**Figure 5:**
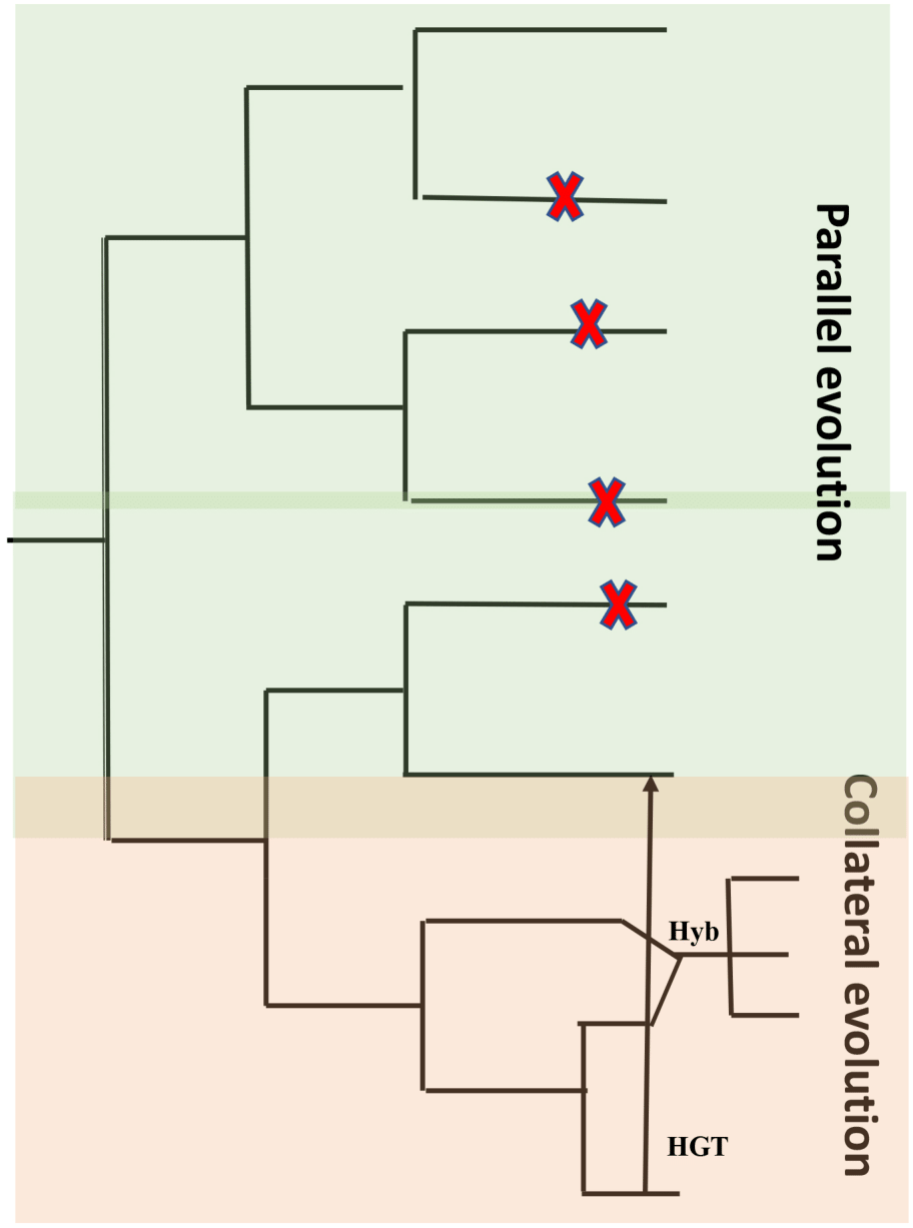
Common types of evolution in obligate pathogens. Parallel evolution (highlighted in green) refer to mutations (X) that arise and evolve in different lineages, resulting in similar/identical phenotypes. In collateral evolution (highlighted in orange), through hybridization (Hyb.), a mutation arises in one lineage and spreads via hybridization to other related species. Another possible mechanism of collateral evolution is horizontal gene transfer (HGT) (indicated as black arrow) that enables the transfer of genes across boundaries of phylogenetic domains. Figure adapted from Kemen, Agler & Kemen (2015).

A characteristic feature of obligate pathogens is the ability to inhibit recognition by the host and suppress host defense mechanisms. This characteristic feature developed independently in distantly related clades of fungi and oomycetes (Thines & Kamoun, 2010; Kemen & Jones, 2012). Characteristic features like host entry and haustoria formation are brought about through similar changes within the genome and the proteome. Another characteristic is a lack of lytic enzymes found in obligate biotrophic filamentous fungi and oomycetes (Kemen, Agler & Kemen, 2015). Similar characteristics could be the result of identical genetic changes caused by mutations that are identical in independent decedents from a common ancestor (parallel evolution) or by introducing an allele from a line into another related line by hybridization (Stern, 2013) or horizontal gene transfer (HGT) (collateral evolution) (Fig. 5). With the availability of genomic sequences, it may be argued that all of them contribute to observed convergence. Characteristic biotrophic adaptations of obligate parasites for existence inside plant cells are as follows.

#### Parallel evolution

Parallel evolution is the independent evolution, from a common ancestor, of similar characteristics between two different species due to similar environments or other evolutionary pressures. Parallel evolution is commonly seen in more closely related lineages where several species handle similar challenges in the same way. In the case of obligate parasites, similar genetic changes can be the result of similar or identical mutations in independent lineages. In summary, parallel evolution emerges after strong selection and suggests limited avenues for mutational adaptation to a specific environmental condition (Bailey et al., 2017).

Here are some examples that explain parallel evolution in obligate pathogens not due to a gain in gene function but due to gene loss. Although this is debatable if gene loss is responsible for evolution in obligate pathogens, there are examples to validate the same. Of interest is the obligate human pathogen *Pneumocystis jirovecii,* known to have acquired obligate biotrophy through gene loss. This was validated by comparative genome analysis (Cissé, Pagni & Hauser, 2014). Gene loss can result from insertion of transposable elements, gene loss via deletion (sometimes after gene disruption), and is also known to drive the evolution of obligate pathogenic bacteria (Bryant, Chewapreecha & Bentley, 2012). Host microbe interaction is associated with effector proteins that alter the host’s metabolism and suppress the host defense mechanisms (Panstruga & Dodds, 2009). Gain of species-specific variability in effector proteins increases pathogenicity (Kemen & Jones, 2012). The Effector P 2.0 program (Effector P2.0, is a machine learning classifier for fungal effector prediction) works on a large set of effectors and is based on an ensemble of classifiers trained on different subsets of negative data, providing different viewpoints on classification. Effector P2.0, available at http://effectorp.csiro.au/, has made a prediction that 12% of the proteins secreted by saprophytes are effector-like (Sperschneider et al., 2018). Comparative genomic analysis in obligate pathogens and in saprophytes has unveiled a fascinating role of the collections of effector molecules. Genome-wide studies on how pathogenic lifestyles were achieved by these obligate pathogens validated more microorganism groups that were subspecies (SSPs) specific than groups that were non-pathogenic (Seidl et al., 2015). There were a class of effectors that were associated with the pathogenic group of microorganisms and were probably associated with interaction with the host. However, there was another class that was conserved within the saprophytes (Zhang et al., 2018). Hence, it can be concluded that some effectors are specific to host microbe interaction in pathogenic species, however there are others designated as “effector-like” proteins that were seen in the saprotrophic pathogens and their function with obligate pathogens have yet to be understood.

Because the salicylic acid (SA) pathway is a common immune strategy utilized by hosts to thwart biotrophic plant pathogens, these pathogens have adapted effectors to prevent the accumulation of SA. *Ustilago maydis* and *Hyaloperonospora arabidopsidis* are two examples of unrelated pathogens whose effectors Cmu1 (a chorismite mutase) and HaRxL44 (a nuclear-localized effector) work to regulate the SA pathway within host plants. Synthesis of the host hormone, salicyclic acid (SA), is prevented by Cmu1 via the effector’s action of re-channeling host chorismite metabolism. The effector HaRxL44 works by shifting the plant’s immune response from the SA pathway to ethylene and Jasmonic acid (JA) immune defense pathways, making the host plant more susceptible to biotrophic pathogens (Mukhtar et al., 2011; Ökmen & Doehlemann, 2014). It has been proposed that parallel evolution in obligate pathogens is the result of gene duplication and diversification because of a gain in gene function. However, it has also been suggested that evolution in obligate biotrophs is not due to gene loss, rather it is dependent on haustorium development. The essential step required for biotrophy is probably a defense suppression mechanism to facilitate efficient functioning of haustoria; subsequent loss of biosynthetic pathways is likely to be secondary (Kemen et al., 2011) (Fig. 2).

In both fungi and oomycetes, the evolutionary pathway which allows for survival in a living host cell is because of the loss of metabolic pathways (Kemen et al., 2011). This may be seen through the way in which metabolic enzyme production decreased (*e.g.*, for thiamine and molybdopterin biosynthesis) and carbohydrate-active enzyme production reduced (Tisserant et al., 2013). Nitrate and nitrite reductases, a nitrate transporter, or sulfite reductase were also reduced (Fig. 3). The absence of certain pathways in obligate biotrophs indicates that convergent loss was linked to selective advantage (Kemen, Agler & Kemen, 2015). A possible explanation for this could be that when the same function or metabolite was associated with the host plant, the energy cost was significantly reduced (*e.g.*, thiamine, molybdopterin, sulfite oxidase, nitrate oxidase) (Morris, Lenski & Zinser, 2012). Comparative genomic studies between closely related dicot-infecting smut fungi *Melanopsichium pennsylvanicum* and *U. maydis* and other monocot-infecting smuts show that they possess similar core eukaryotic genes, but *M. Pennsylvaniancum* also lacks some secreted proteins (Kemen, Agler & Kemen, 2015). Based on the information, to adapt to the dicot host, gene loss was more likely than gene gain (Sharma et al., 2014) only if it is assumed that the fungus was associated with a monocot.

In yet another example, *Pneumocystis jirovecii* (human pathogen) evolved to an obligate biotroph through gene loss. Inorganic nitrogen and sulfur assimilation, thiamine biosynthesis, and purine catabolism accounted for the majority of the functions associated with the genes lost (Cissé, Pagni & Hauser, 2014). This example shows that gene loss is not exclusive to plant pathogens but is also present in animal pathogens that are obligate biotrophs.

### Collateral evolution

HGT is an example of collateral evolution, and it causes a transfer of genes from fungi to oomycete genomes. Although the exact mechanism by which this HGT occurs is unclear, HGT has been demonstrated to be an important part of collateral evolution between fungi and oomycetes (Savory, Leonard & Richards, 2015). In obligate pathogens, the genes transferred encode proteins that attack or feed on plants (Richards et al., 2011). Hemibiotrophic oomycetes are more likely to exhibit HGT than obligate biotrophic oomycetes such as *H. arabidopsidis* and *Albugo laibachii*. *H. arabidopsidis* has been reported to have 21 putative HGTs (representing 3.6% of the secretome); computational validation was possible with only one HGT in *Albugo* (Richards et al., 2011). The HGTs transferred from fungi to oomycetes encode for proteins that assist in sugar degradation, transportation, and reorganization. HGTs from fungi to oomycetes involve genes coding for proteins that function as catalysts in the metabolism, transport, and structural changes in sugars. However, obligate biotrophs do not possess many plant degrading enzymes to avoid defense activation. Because lytic enzymes are harmful for obligate pathogen sustenance, it will not be in the best interest of the pathogen if these genes are transferred. Effectors with no known function and origin are race-specific and usually are not functional in other organisms.

Oomycetes that possess diverse families of Nep1-like proteins (NLPs) which are species-specific have undergone one HGT event and experienced considerable divergent selection (Soanes & Richards, 2014). As well as causing necrosis in certain host species, NLPs can contribute to virulence and disease (Gijzen & Nürnberger, 2006). A comparison between downy mildews and *Phytophthora* revealed NLPs are reduced in downy mildews, with regards to Phytophthora (Baxter et al., 2010). The 12 NLP-coding genes found in downy mildew are not responsible for necrosis (Cabral et al., 2012). In contrast to its hemibiotrophic relative, the biotrophic pathogen’s proteins appear to have evolved over time (Baxter et al., 2010). This also suggests a possible role in microbe–microbe interaction. In gnotobiotic systems, the function of NLPs as environmental factors can be determined by using downy mildew NLP-knockout mutants. By HGT, a virulence factor (*e.g.*, NLP) introduced between populations can give rise to identical phenotypes (collateral evolution), however over time the development of these new functions may cause confusion regarding the original similarity (Fig. 5).

### Host jumps to escape environmental pressure

Host jumps are essential for pathogen survival in a host. The phenomenon is brought about by effector molecules which allow for infection of and survival within another host. This is seen when the phylogenetic distance is short among the two hosts with similar effector targets. This can also be seen in the hemibiotrophic lineage *Proteus mirabilis* (Dong et al., 2014).

A drastic change, such as gene loss, is required if the jump is made from a monocot plant host to a dicot plant host as in the case of *Melanopsichium pennsylvanicum* (Sharma et al., 2014). A bigger jump to a host that is very distantly related happens when the defense mechanism of the new host is suppressed in relation to the original host. When susceptibility does not occur, natural infection can be observed in a nontraditional host. This occurs when the pathogen silences its own defense mechanism (Cooper et al., 2008). Host jumps happens when the immunity of the host is in question and when induced susceptibility is a natural phenomenon. An example is spore dispersal to a dead nearby host (Kemen, Agler & Kemen, 2015). This is an example of an environmental change that leads to temporary infection abilities which help in host jumps by the pathogen (Antia et al., 2003).

Compatibility of pathogens leads to susceptibility of the isolates that were not compatible at first (Ouchi, Oku & Hibino, 1976; Heath, 1980). Several studies have confirmed this in rust and powdery mildew fungi. If plants are co-infected, their susceptibility is limited to a few cells away from the primary infection site. Under such conditions, reproduction may not be possible (Kemen, Agler & Kemen, 2015). The induced susceptibility of oomycetes between *A. candida* and *H. arabidopsidis* has been studied as well.* A.candida* and *H. arabidopsidis* co-infect *Brassica* sp. naturally. When appropriately pre-infected, *A. candida* greatly enhanced the disease-causing ability of compatible *H. arabidopsidis*, but *Albugo* showed a lower multiplication rate (Singh et al., 2002). An infection with a non-sporulating *H. Arabidopsidis* was caused by rapid spore formation caused by virulent* A. candida* (Kaur et al., 2011). Isolates of the same species that are incompatible cannot cause susceptibility to *A. candida* (Singh et al., 1999). Thus, susceptibility that is induced and not natural is efficient among microbes that utilize the same target effectors or other resources. There has also been evolution to limit resource competition among obligate biotrophs, perhaps through effector mediated relationships.

### Role of transposable elements (TEs)

TEs have a role in evolutionary changes that lead to pathogenicity and survival ability (Manning et al., 2013). They are also responsible for gain and loss of gene function, chromosomal rearrangements, and complete inactivation of genes (Biémont, 2010). Expansion in genome size, alternative splicing and exonization, alteration of gene expression, alteration of a regulatory network, epigenetic control, and TEs all contribute to genome plasticity. Genome plasticity enables organisms to adapt to environmental changes. Adaptive evolution mediated by TEs is facilitated by recombination events resulting in genomic diversification. This is achieved through genomic changes which persist under positive selection in obligate fungal pathogens.

TEs induce pathogenicity by their proximity with avirulence/pathogenicity associated genes. TEs are known to gain virulence through deletion of avirulence genes, and they promote pathogenicity by inducing nucleotide diversity. Mutations from TE insertions can lead to genetic variability that generates many new pathogenic variants with conferred ability to invade previously resistant host plants (overcome host plant resistance) and hence expand on the host range. TEs are known to alter host fitness through deleterious insertions, driving speciation and adaptive evolution.

The occurrence of many TEs in wheat biotrophs had led to a rapid evolution of the genome (Amselem, Lebrun & Quesneville, 2015). As an example, we have seen an expansion of the genome size of rust fungi, often between 100 and 200 Mb (Cuomo et al., 2017). Most genome sizes of other basidiomycete fungi are less than 50 Mb (Duplessis, Bakkeren & Hamelin, 2014). The larger size is mainly contributed to from high amounts of repetitive DNA referred to as TEs. A *Pst* race PST-130 has TEs which contribute to 17.8% of the genome. The genome of the Chinese isolate of CYR32 has 50% TEs, hence the genome size almost twice as large (Zheng et al., 2013; Cuomo et al., 2017). A member of the Puccinia genus, *Pucciniatriticina* (*Pt*), had a higher repeat content, with an average of more than 51% (Cuomo et al., 2017). The *Bgt* genome had even more TEs, reaching 90% (Wicker et al., 2013).

### Role of secreted proteins

Obligate biotrophic pathogens are associated with secreted proteins (SPs) that help with escaping host immune responses (Fig. 4). Species-specific genes coding for SPs were found in the genomes of rust and powdery mildew. The *Pst* genome contains 2092 SPs, which account for 8.3% of the total number of predicted protein genes (Zheng et al., 2013). Two *Pt* races produced 660 SPs (Kiran et al., 2016), while a member of the Puccinia genus, *Pgraminis* f.sp. *tritici* (*Pgt*), produces 1459 SPs (Zheng et al., 2013).

A virulent *Pt* isolate showed more SPs than race-specific isolates with a narrow virulence scope. These SPs are unique to each species of the pathogen because of the fast-evolutionary modification of the protein. The rapid evolution of some effectors indicates their individual pathogen specificity. As discussed previously, of the three rust fungi, 62% of SPs are unique to that species. It has been shown that 5% of SPs are found only exclusively in the rust and powdery mildew fungi (Spanu et al., 2010; Dean et al., 2005), indicating that each pathogen evolved differently.

### Role of haustoria

Haustoria (Fig. 1B) are associated with effector delivery and nutrient uptake (Voegele et al., 2001). RNA transcriptomic studies of *Pgt* haustoria and germinated urediniospores showed genes that were upregulated in germinated urediospores. These genes were associated with cell proliferation, cell wall synthesis (Upadhyaya et al., 2014), and DNA replication. The haustorium is crucial for biotrophic colonization of *Pst* as it enhances expression of genes involved in ATP and TCA synthesis (Garnica et al., 2013). A total of 520 secreted proteins (HSPs), 430 upregulated secreted proteins in haustoria, and 90 genes were identified for *Pgt* (Cuomo et al., 2017). To identify specific avirulence alleles whose transcripts bind resistance genes, the effectors must interact with their corresponding targets (Flor, 1959; Moseman, 1959). Rust and powdery mildew produce effectors in haustoria, which are then transferred to host cells. Novel effector haustorial proteins are described here (Elmore et al., 2020; Garnica et al., 2014; Link et al., 2014; Lorrain et al., 2019; Polonio et al., 2019; Tao et al., 2017). *U. fabae’s* haustorium expressed the rust transferred protein 1 (RTP1), which translocated into the cytoplasm of the host during the interaction (Lawrence, Dodds & Ellis, 2010). Stripe Rust CYR31 race haustoriums contained 1,197 secreted proteins, 69 of which inhibited tobacco cell death and 49 of which suppressed wheat callose deposition. Transcriptomic studies were used to identify these proteins. Infection processes are associated with these proteins in *P. striiformis* (Xu et al., 2020). It is possible to further screen these effector proteins identified by haustorial studies for features associated with avirulence.

### Role of effectors

There have been no definitive studies determining whether rust fungi effectors play a role in pathogenicity. In rust pathogens, there were no knockout mutants available. Small interfering RNAs (siRNAs) obtained from pathogen dsRNA can be expressed in plants. These siRNAs were able to enter the pathogen and silence their transcripts (Jaswal et al., 2020), which is referred to as host-induced gene silencing (HIGS). Fungi, oomycetes, and insects have been shown to exhibit this phenomenon. Based on this information, a Barley stripe mosaic virus (BSMV)-mediated HIGS system was created to silence any *Pst* genes, by expressing dsRNAs derived from Puccinia (Yin, Jurgenson & Hulbert, 2011). It has been demonstrated that BSMV-HIGS inhibited the expression of haustoria-specific genes. In plants, compromising *Pst* infection also led to silencing effectors (PEC6 and PSTha5a23) (Yin, Jurgenson & Hulbert, 2011; Cheng et al., 2017). This model system helps evaluate rust pathogen effectors via transient silencing.

The rust fungal pathogens are known to contain eight effector proteins, including RTp1 which was transferred from rust pathogens and obtained from *Uromyces fabae*, four effector proteins from *M. lini, AvrP4, AvrM, AvrL567* and *AvrP123*, and three effectors *PGTAUSPE-10-1*, *Avr35* and *Avr50* from *P. graminis* (Cheng et al., 2017; Petre, Joly & Duplessis, 2014; Prasad et al., 2019; Salcedo et al., 2017). Two additional *AVRs* were seen in *P. graminis: Avr35* and *Avr50. P. graminis* mutants and non-mutant isolates were analyzed using comparative genetics in order to identify *AVRSr35* associates with *Sr35* (wheat resistance gene). A mobile element inserted into the *AvrSr35* gene altered functional characteristics which also included susceptibility (Salcedo et al., 2017). Another study was able to demonstrate the interaction between the avirulence gene-encoding protein AvrSr50 secreted by haustoria cells and the immune receptor Sr50. AvrSr50 originated from a naturally occurring mutant of *P. graminis* with a 2.5 megabase pair deletion in its genome. As a result of these groundbreaking studies, susceptibility factors of pathogens have been identified (Cheng et al., 2017). Genome-wide association studies (GWAS) and map-based cloning have identified nonvirulent genes that have structural resemblance to RNase-like proteins seen in wheat and rye powdery mildew pathogens (Praz et al., 2017).

Studies on effector molecules in powdery mildew detected an association between (*Mla*) genes and barley mildew resistance. These genes have the functional capability to identify effectors from a wide variety of gene families (Jaswal et al., 2020).

### Role of small RNA

It is believed that small noncoding RNA molecules, such as microRNA, regulate vital functions in the host (Dubey et al., 2019; Kusch et al., 2018; Mueth, Ramachandran & Hulbert, 2015; Wang et al., 2017a; Weiberg & Jin, 2015; Jaswal et al., 2020). They regulate genes associated with defense mechanisms and immunity at different developmental stages with a functional role similar to effector proteins. It has been shown that sRNAs can move in the cytoplasm and silence the host defense genes of *P. striiformis.* This movement happens because of the presence of extracellular vesicles (Wang et al., 2017a; Wang et al., 2017b; Jaswal et al., 2020). Genome-wide sRNA association studies in the host and pathogen indicated that sRNA may inhibit the host’s own effector genes and target any genes associated with immunity, such as RLKs (receptor-like kinase) and NBS-LRR (nucleotide binding site- leucine-rich repeat) proteins. As a result, they can interact with the host similarly to how effector molecules do (Dubey et al., 2019; Sperschneider et al., 2018; Jaswal et al., 2020). The presence of these molecules in wheat leaf rust is seen at different developmental stages (resting spores, germinated spores at 16 and 24 h, and highly infected wheat leaf) demonstrated the presence of multiple defense-related genes, like reactive oxygen species (ROS), transcription factors (RLKs), and any resistant genes associated with diseases (Dubey et al., 2019). In *P. striiformis* (Pstr) there are a wide range of sRNAs that silence any endogenous genes in the host and genes related to defense and immunity (Mueth, Ramachandran & Hulbert, 2015).

### Role of secondary metabolites

The secondary metabolites (SMs) act as non-proteinaceous effectors that manipulate the host with toxins (Castro-Moretti et al., 2020; Collemare, O’Connell & Lebrun, 2019; Pusztahelyi, Holb & Pocsi, 2015). SMs also function as nonvirulent factors and suppressing host defense mechanisms and strengthening cell wall factors (Collemare, O’Connell & Lebrun, 2019; Lo Presti et al., 2015; Pusztahelyi, Holb & Pocsi, 2015). By inducing penetration of the fungal cell, SMs are primarily engaged in biotrophic infection, causing infection to take place without killing the host. There has been an increase of SM production reported in genome and transcriptomic studies during different developmental stages of pathogen development in the host (Collemare, O’Connell & Lebrun, 2019; Keller, 2019; Rokas, Wisecaver & Lind, 2018).

### Role of transporters

During infection, the transporter gene family was upregulated (Fig. 4). When rust pathogenesis is triggered, hyphae from haustoria which feed off the plant’s carbohydrates and amino acids at their active functional state (Ellis et al., 2009; El Gueddari et al., 2002; Voegele et al., 2001). Membrane transporters of *M. larici-populina* and *P. graminis* f. sp.* tritici* have homologs of the HXT1, AAT1, AAT2, and AAT3 transporters and H + ATPases from the bean rust pathogen *U. fabae* (Duplessis et al., 2011). Whenever a pathogen interacts with a host, all these transporters are upregulated. *M. larici-populina* and *P. graminis* f. sp. *tritici* exhibit higher levels of peptide uptake due to the presence of 22 and 21 oligopeptide membrane transporter (OPT) genes, respectively. Only 5-16 OPT genes were found in other basidiomycete fungi (Duplessis et al., 2011). OPT genes upregulated *in planta* (Duplessis et al., 2011) transport peptides released by inducible proteases (aspartic peptidase, subtilisin) once the leaf tissues are infected (Fig. 4). A reduction of the Major Facilitator Superfamily (MFS) is observed in *M. larici-populina* and *P. graminis* f. sp. *tritici* genomes when compared with other basidiomycetes (Duplessis et al., 2011). However, many MFS transcripts are upregulated in planta, such as HXT1 homologues. In the plant host expression of *M. larici-populina* and *P. graminis* f. sp. *tritici,* invertase genes are upregulated (Duplessis et al., 2011). Host hexoses, such as sucrose transporter *Srt1, are* typically used by invading rust pathogen hyphae (Voegele et al., 2001). There is no homologue for this sucrose transporter. During the invasion of rust fungi, membrane transporters play a critical role in providing the necessary fuel due to the high metabolic activity (Duplessis et al., 2011). It was also noticed that auxin efflux gene expression was much higher in rust fungi compared to other basidiomycetes (Duplessis et al., 2011). In *U. maydis*, auxin synthesis gene homologs are upregulated during infection of the host (Turian & Hamilton, 1960; Basse et al., 1996; Reineke et al., 2008). The growth of plants depends on auxin synthesis while pathogen auxins are crucial for host signaling, defense strategies, or plant cell wall integrity during rust infections.

### Role of carbohydrate-active enzymes

In addition to proteases, lipases, and sugar-cleaving enzymes, a variety of carbohydrate-active enzymes (CAZymes) are up-regulated (Fig. 4) in rust pathogen plants (Cantarel et al., 2009; Duplessis et al., 2011). This suggests that the pathogen uses degradative enzymes and fungal hyphae to penetrate and enter the host cell. Upon comparing 21 sequenced fungi, it was found that glycoside hydrolases (GH), glycosyltransferases (GT), polysaccharide lyases (PL), and carbohydrate esterases (CE) (Duplessis et al., 2011) are similar to those found in the basidiomycete symbiont, *L. bicolor* (Martin et al., 2008), but are less numerous than hemibiotrophs and necrotrophs (*e.g.*, *Magnaporthe oryzae*), and saprotrophs (including *Neurospora crassa*; *Coprinopsis cinerea*; *Schizophyllum commune*) (Ohm et al., 2010). The biotroph *U. maydis* does not contain many CAZymes (100 members) (Kämper et al., 2006). The evolution of a biotrophic lifestyle in rust fungi resulted in the loss of secreted hydrolytic GH and PL enzymes known to interact with plant cell wall polysaccharides (Duplessis et al., 2011). Evolution to the biotrophic lifestyle also led to the loss of cellulose binding carbohydrate-binding module 1 (CBM1) (Fig. 4). The GHs that cleave plant celluloses and hemicelluloses are moderately upregulated (*e.g.*, GH7, GH10, GH12, GH26, and GH27) in comparison with biotrophs U.* maydis* or *M. oryzae* (which is the hemibiotroph). Plants with these upregulated enzymes have also shown that presence of *α*-mannosidase (GH47) and *β*-1,3-glucanase (GH5) transcripts (Duplessis et al., 2011) are involved with colonization or penetration of parenchymal cells. The cell wall of a fungus is reconstructed and altered to disguise invading hypha from the host when there is an infection (El Gueddari et al., 2002) by chitin deacetylases (CE4), something which is also seen in *P. graminis* f. sp. *tritici*, *M. laricipopulina*, and the symbiont *L. bicolor* (Martin et al., 2008).

### Nitrate and sulfate assimilation pathway deficiencies in rust fungi

Rust fungi, like other obligate pathogens, cannot grow *in vitro*. Hence, *M. larici-populina* and *P. graminis* f. sp. *tritici* are unlikely to carry genes normally found in saprotrophic basidiomycetes. Several anabolic pathways have been scrutinized for possible deficiencies. NH4+ assimilation enzymes are present. However, nitrate assimilation genes were not present in either rust pathogen genome. Nitrate/nitrite porters and nitrite reductases were not found in other fungis’ gene clusters associated with nitrate assimilation (Slot & Hibbett, 2007). Sulfate assimilation genes were found in *M. larici-populina,* but not in* P. graminis f.* sp. *tritici*. SiR subunits, *α*- and *β* of sulfite reductase, were absent in the latter fungus, whereas the *M. larici-populina β*-subunit of SiR has no transketolase domain similar to the SiRs found in other fungal systems. Both rust fungi have dysfunctional nitrate and sulfate assimilation pathways that can be related to obligate biotrophs. This is because they need minimal nitrogen sources (different amino acids and ammonium ion) and there is no uptake of sulfur from the plant system (Duplessis et al., 2011). The same metabolic deficiencies (Fig. 3) have been discovered in different plant pathogens from different evolutionary lineages, one belonging to the oomycete (*H. arabidopsis*) and the other to the ascomycete (*B. graminis*) (Spanu et al., 2010; Baxter et al., 2010).

### Role of signal molecules in obligate parasitism

A new hypothesis suggests signal molecules coming from the host plant helps in an obligate biotrophic lifestyle through the regulation of metabolic gene expressions. (Fig. 3). Infection specific organs, like haustoria and appressoria (specialized structures for nutrient uptake and entry respectively), develop in response to plant signals (Hamer & Talbot, 1998; Tucker & Talbot, 2001). In plants, for example, rusts recognize the surface of the cell wall, prompting hyphae to form (Staples, 2000). In *Magnaporthe*, hydrophobic surfaces cause appressoria to develop (Talbot et al., 1996; Hamer & Talbot, 1998). Products known to degrade cutin cause appressoria formation in Blumeria (Both & Spanu, 2004). It is possible that this essential factor controls the essential metabolic machinery of biotrophs involved in nutrient uptake and utilization. The fungus must reveal its transporters and pumps at the exact moment, at the right locations, and at the correct intensity to survive. Plant derived stimuli, rather than feeding structures, are essential to survival. Furthermore, during their life cycle, it is these stimuli that catalyze the catabolic and anabolic reactions necessary for growth and nutrition of biotrophs. At the moment, the alternative hypothesis is supported by some direct evidence. A variety of metabolic genes are expressed during both development and pathogen attack in barley powdery mildew (Both et al., 2005), as well as functional characteristics associated with uptake in rust haustoria (Sohn et al., 2000; Voegele et al., 2001; Jakupovic et al., 2006). In arbuscular mycorrhizal fungi, intraradical mycelium was found to contain fatty acid synthase activity (Trepanier et al., 2005), and mycelium associated with intraradical and extraradical growth exhibit differential expression of genes related to nitrogenous compound metabolism (Govindarajulu et al., 2005). Based on this new hypothesis, the expression pattern displayed during pathogen germination should be disrupted when fungi are observed to partially grow *in vitro*. Another test will determine if there are deficiencies or mutations in the regulatory elements (*e.g.*, promoters) that control metabolic genes. The whole genomes of obligate and non-obligate related fungi can be compared to unravel this information. There may be a connection between evolutionary radiation and the spontaneous development and modifications in regulatory mechanisms of genes which bring about a major change in the expression pattern (Gilad et al., 2006).

## Discussion

In summary, it can be concluded from the literature that there are adaptative features in both fungi and oomycetes which are essential for maintaining adaptation strategies and pathogenicity of obligate biotrophs. These include:

(i) A very large genome size which is crucial to rapid evolution. For example, two genomes of rust fungi, M. larici-populina (89 Mbp, 16,339 proteins) and P. graminis (101 Mbp, 17,773 proteins), exhibit minimal conservation of gene order. It is possible that recombination between transposable elements (TEs) and TE proliferation is what causes the dynamicity of rust genomes. It has been seen that within rust pathogen genomes there are an increased number of gene families which encode DNA repair enzymes. Possessing a fluid genome could allow for a pathogen which lives solely within a host to rapidly adapt, allowing for the continuation of the pathogen’s survival within the host. A total of 65% of *P. graminis* predicted proteins, and 59% of *M. larici-populina* predicted proteins are not available in the Genbank database, showing that rust genomes possess unique genes. Furthermore, lineage-specific expansions of the gene families abundantly transcribed during infection show gene families that are specific to rusts. Future studies should examine these rust-specific gene families to develop an deeper understanding of the adaptations which may enhance a host-specific obligate lifestyle (McDowell, 2011).

(ii) Uptake and assimilation of organic nitrogen and sulfur from host sources. In the case of downy mildews, these pathogens have lost enzymes which allow them to assimilate inorganic nitrogen and sulfate. Additionally, powdery mildews are also unable to assimilate inorganic nitrogen due to a loss of enzymes. Compared to related pathogenic ascomycetes, the genomes of powdery mildews are larger by over fourfold. Among pathogenic oomycetes, the genomes of downy mildews are some of the largest. Both the genomes of powdery mildews and downy mildews have a high percentage of transposable and repeated elements, as well as a high number of lineage-specific genes (McDowell, 2011).

(iii) Loss of genes to suppress host immune responses. When compared to other necrotrophic species, downy and powdery mildews have a substantial reduction in activators of host defense, such as reduced secreted degradative enzymes. Mechanisms which allow the pathogen to avoid the host’s defense are pervasive, and likely necessary among obligate pathogens. It is furthermore necessary for obligate biotrophs to maintain the viability of their host cell throughout the pathogen’s life. The host’s defenses may be activated not only due to signals released from the pathogen but may also be activated due to an altered host cell status (McDowell, 2011). In rusts, genes encoding proteins which may harm the host cell are reduced, such as genes encoding carbohydrate active enzymes and secreted toxins. However, in rusts there is an expansion of chitin deacetylases which help interfere with the fungal cell wall’s structural component, chitin, as chitin may activate the host’s defense mechanisms. These adaptations of rusts allow for their ability to remain undetected within the tissue of their host plants (McDowell, 2011).

(iv) An array of secreted effector proteins functioning inside and outside of host cells to build immunity and initiate survival within a host. Evidence indicates that host immunity may be interfered with by oomycete effectors, such as can be seen in the case of downy and powdery mildews which contain genomes encoding for a large number of candidate secreted effector proteins. The effecterome of P. graminis encodes for 1,106 small secreted proteins (SSP)s, and the effecterome of M. larici-populina encodes for 884 SSPs. Interestingly, over one-half of these SSPs are transcribed during infection of the host plant, 16% of which are conserved between *P. graminis* and *M. larici-populina*. This may be an indication of rapid turnover, allowing these pathogens to either evade the host’s immune surveillance or to interact with new host targets (McDowell, 2011).

These findings can improve current strategies for plant breeding with stable resistance.

## Conclusions

Genomic and transcriptomic studies have allowed us to conclude that the evolution of biotrophy is a multistep process (Duplessis et al., 2011). Characteristic features in the evolutionary pathway include (1) progressive development of effectors to assist in defense, (2) attenuated activation of defense by decreasing cell wall hydrolyzing enzymes, resulting in, (3) certain biosynthetic pathways functioning poorly if their reactants are obtained from the host. Eventually, this process leads to irreversible biotrophy due to progressive auxotrophy.

Information related to the obligate biotrophic lifestyle can be obtained by sequencing the genomes of wheat stem rust fungi and poplar leaf. Scientists are unsure exactly how nonbiotrophic progenitors evolved into obligate biotrophs. Comparisons of obligate biotrophs *M. larici-populina,* and *P. graminis* f. sp. *tritici,* to other saprotrophic, pathogenic, and symbiotic basidiomycetes did not show any alteration in the conserved regions of the proteins of the rust fungi with different lineages. However, changes in oligopeptide membrane transporters, auxin efflux carriers, copper/zinc superoxide dismutase, and signaling elements may have resulted from modifications of this pathogen to a more parasitic lifestyle (Duplessis et al., 2011). Also, the zinc finger proteins in the two fungi, seen during plant-pathogen interaction and contributed by transcription factors, do not follow the traditional transcriptional functioning seen in other cases (Duplessis et al., 2011).

Modifications of gene content in these pathogens largely revolves around sets of wider gene families that have a very specific lineage. These gene families assist in structural and functional adaptation.

Lineage-specific proteins enable accumulation of the pathogen in the host leaf, cellular differentiation of pathogenic structures, and plant immune system regulation (Duplessis et al., 2011). These obligate pathogens have evolved and adapted to the plant’s immune system by producing candidate effectors like the SSPs. Gene loss and genome compaction result in the development of bacterial biotrophs and microsporidium fungal parasites (Haas et al., 2009; Levesque et al., 2010), but with rust pathogen genomes, it is different. Because of the large gene families and abundance of TEs, rusts possess one of the largest fungal genomes. There have been no significant gene losses in *M. larici-populina* and *P. graminis* f. sp. *Tritici*. However, gene losses with no major impact have been noticed, such as in the case of nitrate and sulfur assimilation.

In rust fungi and all biotrophic pathogens, gene losses with major impact were observed, such as a reduction in enzymes that degrade polysaccharides (Thines & Kamoun, 2010; Holub & Beynon, 1997). It might be beneficial to understand how factors like SSPs (that are effector like) could affect coevolution and host-pathogen interactions in agricultural and forest ecosystems in order to have efficient parasite-control methods.

## Additional Information and Declarations

### Data Availability

The following information was supplied regarding data availability: This is a literature review article.
